# Response to Letter to the Editor: ‘Don’t work more than 3 days straight’: Working conditions as learning conditions in general practice

**DOI:** 10.1016/j.clinme.2026.100659

**Published:** 2026-10-17

**Authors:** Leonard Grant

**Affiliations:** School of Medicine, University of Liverpool, United Kingdom

Dear Editor,

Thank you for the opportunity to read and engage with the letter sent to you in response to my paper.^1^ I think the authors have landed on a significant insight, one I began to excavate in my work but did not explore to the extent they offer. My participants did not speak of collective approaches to managing their working conditions, certainly not any that would amount to workplace organising. I think that a future project of the nature suggested by the letter writers would be valuable; I did not explore extensively any other factors which could have meant a portfolio career was desirable. I would not wish to overstate the causal nature of my findings; my participants did perceive nine sessions a week to be unsustainable and most, if not all, had a portfolio career of some description, but this does not mean that there were not other reasons for that decision. Future work considering this interplay would be illuminating.

I am also grateful to the letter writers for extending my argument in such a productive way; I absolutely agree with their suggestion that we should ask what the workplace is teaching doctors about their capacity to change it. Outside this research project, I speak only from anecdote; but I do not often hear collective responses to untenable conditions discussed.

The suggestion made to include in education the confidence to identify and challenge the structures that underpin the conditions is excellent and I would be delighted to see this incorporated broadly. Certainly, this is part of the conversation I have with students when I am teaching; the pleasures and difficulties of medical practice do not occur in a vacuum, they are irreducibly material. Just this week, I was facilitating an undergraduate workshop concerned with the qualities of the good doctor. As they discussed their thoughts, I turned the conversation towards the ways in which the structures constrain or allow these various qualities - which are made easy, which are difficult and which are rendered impossible - and how those structures may change.

## CRediT authorship contribution statement

**Leonard Grant:** Writing – original draft, Writing – review & editing.

## Funding

This research did not receive any specific grant from funding agencies in the public, commercial, or not-for-profit sectors.

## Declaration of competing interest

The author declares that they have no known competing financial interests or personal relationships that could have appeared to influence the work reported in this paper.
